# Supplementary material for: Temporal trends in evidence supporting novel drug target discovery
Source: Nat Commun. 2025 Dec 7;17:492. doi: 10.1038/s41467-025-67180-y (PMC12804190; doi:10.1038/s41467-025-67180-y)

## SUPPLEMENTARY INFORMATION

**Supplementary Table S1. Timestamps of evidence supporting target-disease associations in the Open Targets Platform.** Distribution of the number and percentage of dated evidence across data resources. References to the source of the timestamps are provided.

| Source | Evidence | Dated evidence | % of evidence | Timestamp reference |
|---|---|---|---|---|
| Europe PMC | 21,641,315 | 21,641,315 | 100% | a |
| GWAS associations | 1,082,677 | 1,032,949 | 95% | m |
| IMPC | 1,175,688 | 1,015,743 | 86% | b, c |
| Cancer Gene Census | 82,754 | 66,008 | 80% | b, c |
| UniProt curated variants | 33,047 | 32,202 | 97% | b, c |
| GEL PanelApp | 34,784 | 26,329 | 76% | b, c |
| Expression Atlas | 229,404 | 228,676 | 100% | b, c |
| Reactome | 10,162 | 9,839 | 97% | b, c |
| Orphanet | 6,293 | 5,053 | 80% | b, c |
| UniProt literature | 6,714 | 6,646 | 99% | b, c |
| Gene signatures | 390 | 390 | 100% | b, c |
| Cancer Biomarkers | 1,300 | 987 | 76% | d |
| ClinVar | 3,138,383 | 3,128,149 | 100% | e |
| ClinVar (somatic) | 9,719 | 9,712 | 100% | e |
| Gene2phenotype | 4,181 | 3,833 | 92% | f |
| Clingen | 3,016 | 2,952 | 98% | g |
| ChEMBL | 573,124 | 485,135 | 85% | h |
| SLAPenrich | 72,406 | 72,406 | 100% | i |
| Gene Burden | 36,805 | 36,372 | 99% | j |
| CRISPR Screens | 21,711 | 14,226 | 65% | k |
| Project Score | 517 | 517 | 100% | l |
| IntOGen | 4,224 | - | 0% | |
| PROGENy | 378 | - | 0% | |
| All | 28,168,992 | 27,819,439 | 99% | |

a. http://ftp.ebi.ac.uk/pub/databases/opentargets/platform/23.06/output/etl/parquet/evidence/sourceId=europepmc/.

b. https://ftp.ncbi.nlm.nih.gov/pubmed/baseline (last accessed on 24th July 2023).
c. https://ftp.ncbi.nlm.nih.gov/pubmed/updatefiles (last accessed on 24th July 2023).
d. https://www.cancergenomeinterpreter.org/2018/data/cgi_biomarkers_latest.zip.
e. https://gist.github.com/DSuveges/48611eead49ae1bd9553516d72efe8e0.
f. http://ftp.ebi.ac.uk/pub/databases/gene2phenotype/28_04_2023.
g. https://search.clinicalgenome.org/kb/gene-validity/download.
h. http://ftp.ebi.ac.uk/pub/databases/opentargets/platform/23.06/output/etl/parquet/evidence/sourceId=chembl/.
i. Iorio 2018
j. Karczewski 2022. Gene Burden projects, a manifold of whole-exome sequencing analysis on UK Biobank individuals.
k. Tian 2021. CRISPRbrain database with functional genomics screens in differentiated human brain cell types.
l. Behan 2019. Project Score aiming at identifying dependencies in cancer cell lines to guide precision medicine.

https://platform-docs.opentargets.org/gentropy/locus-to-gene-l2g.

**Supplementary Figure S1. Overlap of novelty peaks of target-disease associations supported by multiple data resources.** Each target-disease association has been mapped to the highest scoring novelty peak of each supporting resource and a pairwise correlation analysis has been performed between their onset years. Novelty peaks derived from genetic content resources are the most correlated. This phenomenon can be attributed to the fact that public biomedical data is captured in a variety of repositories and databases, many of which are subsequently incorporated into the Open Targets Platform, often leading to data redundancy. GEL PanelApp, Genomics England PanelApp. OT Genetics, Open Targets Genetics. IMPC, International Mouse Phenotype Consortium.

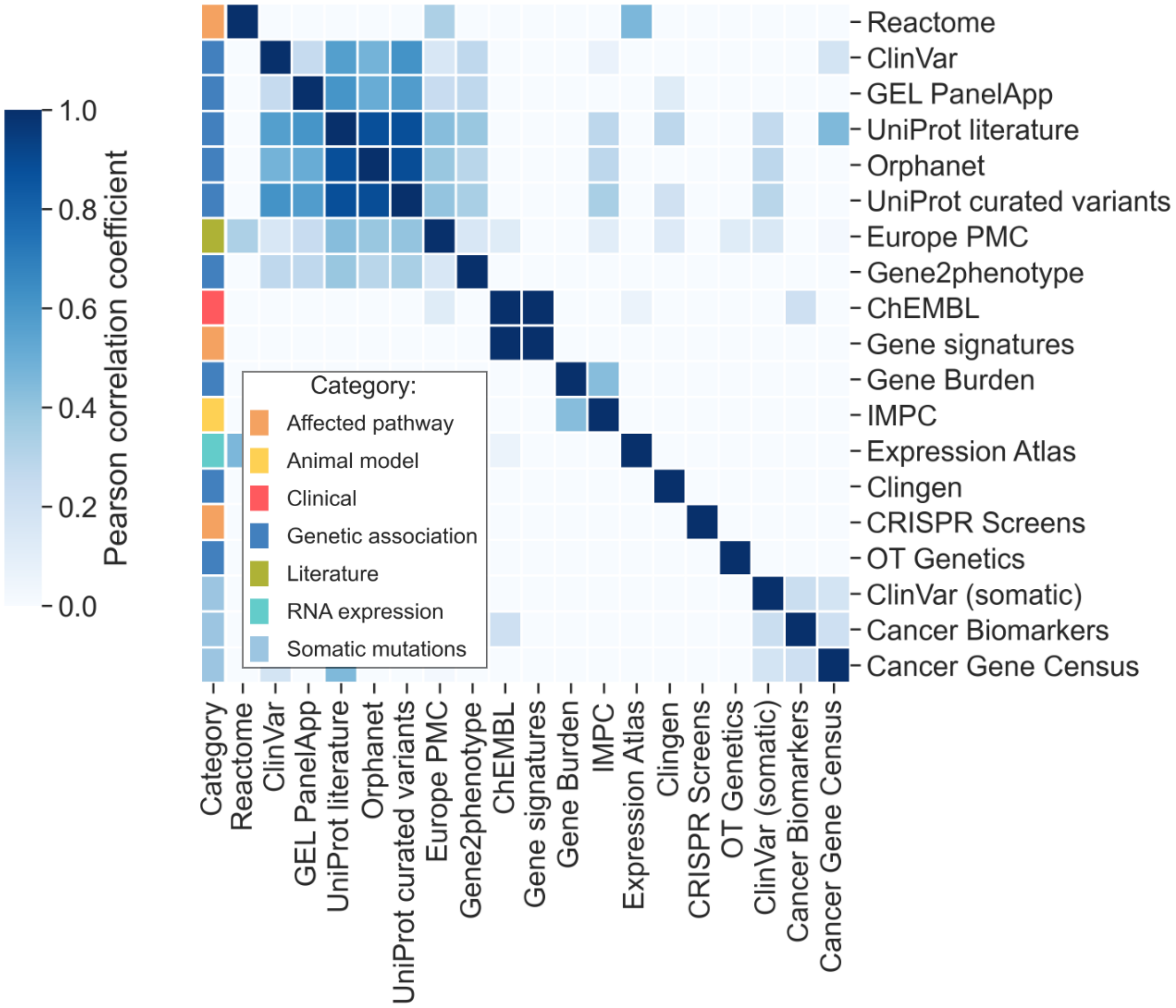

**Supplementary Figure S2. Temporal profiles for the association between thymic stromal lymphopoietin (TSLP) and asthma.** a. The ‘Evidence’ graph shows pieces of evidence after disease otology propagation supporting the association, mapped to their Open Targets Platform evidence score (y-axis), timestamp (x-axis) and source (colour). The ‘Association’ and ‘Novelty’ graphs show how the Platform’s source and overall association and novelty scores have evolved over time. b. Examples of evidence that have triggered shifts in the Platform’s association scores and novelty peaks. The identifiers of the reference clinical trials (NCT) and PubMed Central (PMC) publications are shown.

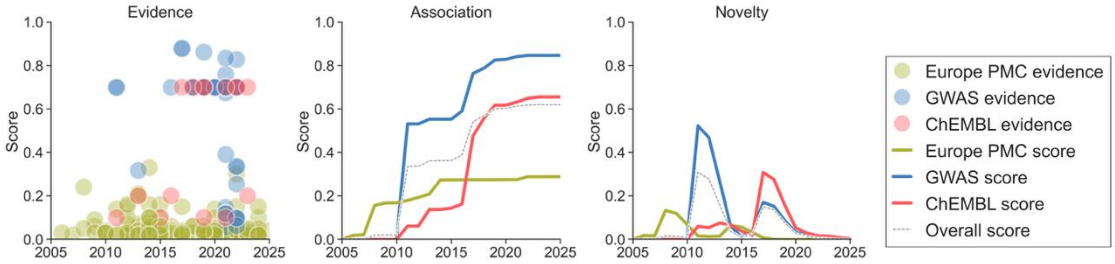


**Supplementary Figure S3. Number of target–disease associations (stacked bar plot) and unique targets (line plot) with novelty peak across data sources over the years.** Associations are assigned to the year in which the highest novelty peak has been reported in each category. Targets are assigned to the first year in which an association involving them has been reported as novel in each category. Bar plots in this figure are a single aggregated count without individual-level data, hence, no individual points exist for the aggregate.

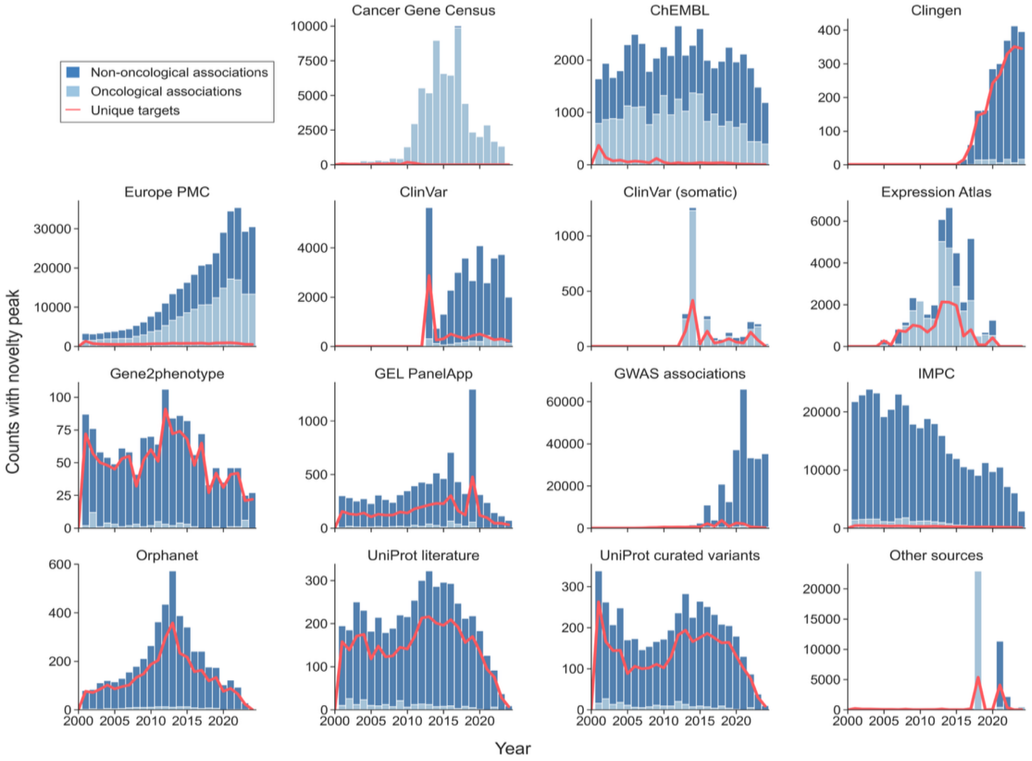

**Supplementary Figure S4. Number of target–disease associations (stacked bar plot) and unique targets (line plot) with novelty peak across data sources over the years.** Associations are assigned to the year in which the highest novelty peak has been reported in each category. Targets are assigned to the first year in which an association involving them has been reported as novel in each category. Evidence have been propagated across the disease ontology. Bar plots in this figure are a single aggregated count without individual-level data, hence, no individual points exist for the aggregate.

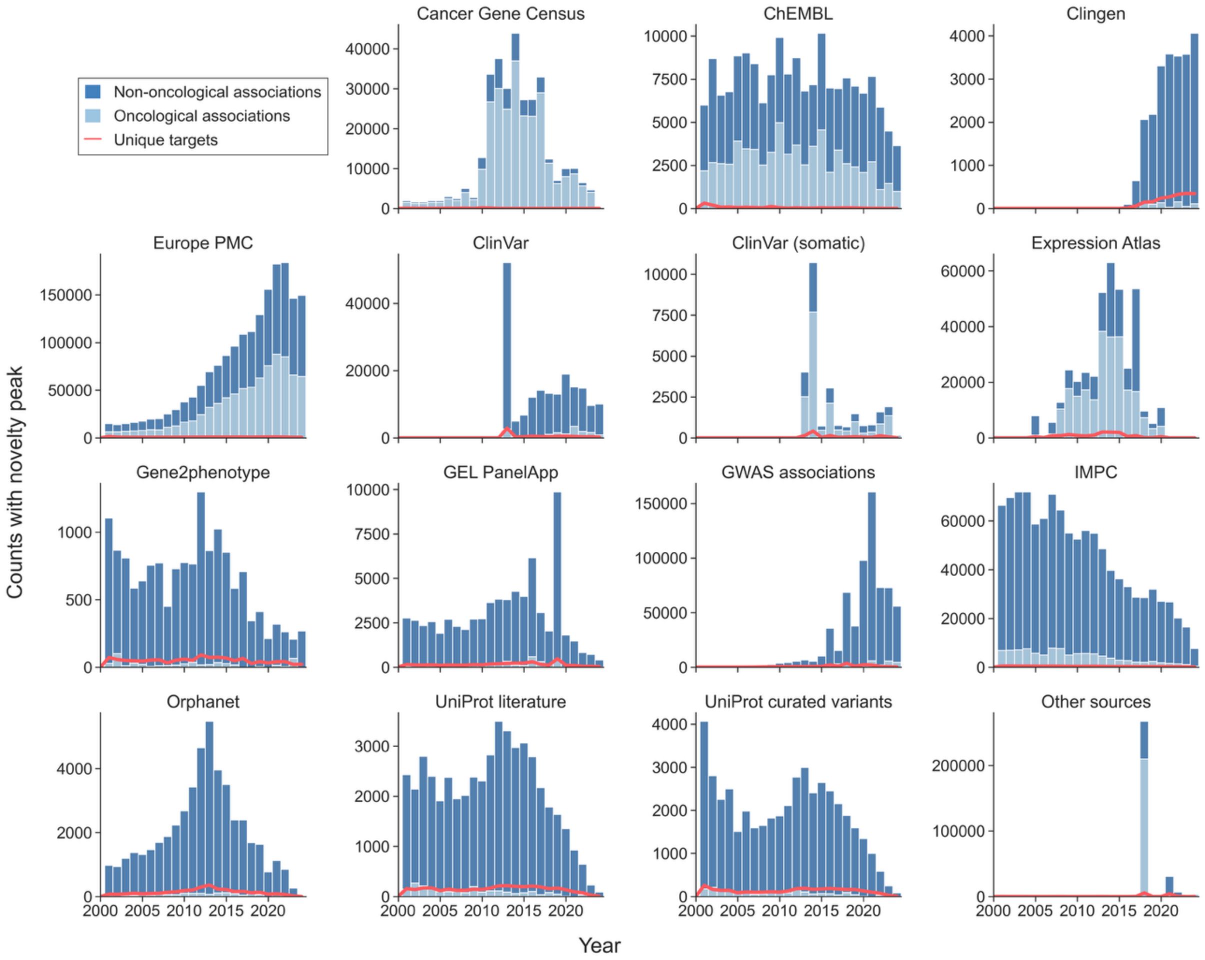

**Supplementary Figure S5. Temporal trends in novel drug target discovery in the last decade**. On the left, mechanism-of-action targets whose first annotated drugs were approved between 2000 and 2005. On the right, mechanism-of-action targets whose first annotated drugs were approved between 2020 and 2025. The window of years elapsed from approval to novelty peaks' onset is shown in the x-axis, and the proportion of drug targets found in each window is shown in the y-axis. Genetic association and somatic mutation peaks are considered together as human genetic data. Clinical peaks have been deconvoluted into clinical phase I/II, III and IV. Bar plots in this figure are a single aggregated count without individual-level data, hence, no individual points exist for the aggregate.

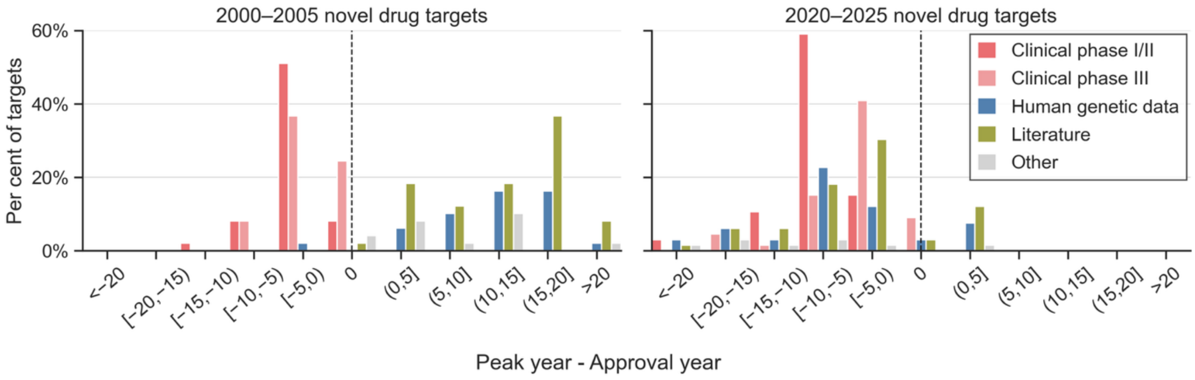


**Supplementary Figure S6. Temporal trends in novel drug target discovery in the last decade**. On the left, mechanism-of-action targets whose first annotated drugs were approved between 2000 and 2005. On the right, mechanism-of-action targets whose first annotated drugs were approved between 2020 and 2025. The window of years elapsed from approval to novelty peaks' onset is shown in the x-axis, and the proportion of drug targets found in each window is shown in the y-axis. Genetic association and somatic mutation peaks are considered together as human genetic data. Clinical peaks have been deconvoluted into clinical phase I/II, III and IV. Supporting evidence have been propagated across the disease ontology. Bar plots in this figure are a single aggregated count without individual-level data, hence, no individual points exist for the aggregate.

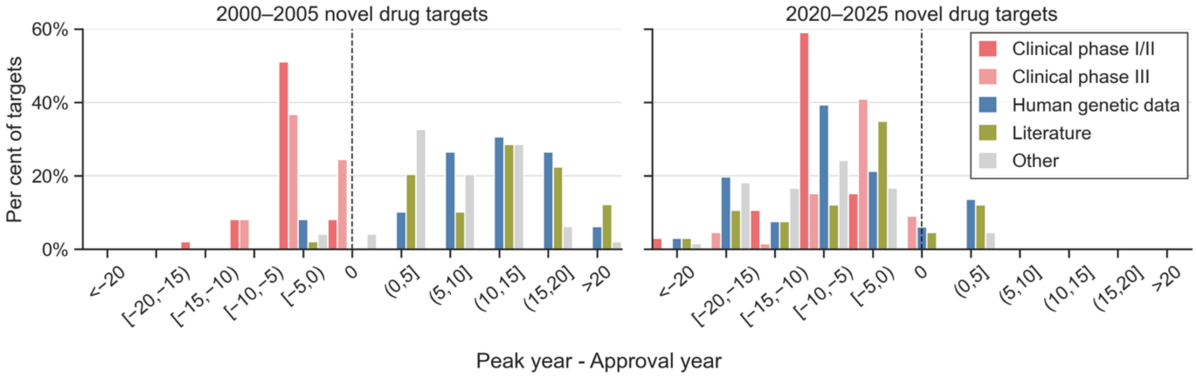

**Supplementary Table S2. Number of novel drug targets since 2000 with support across data resources**. Direct support and indirect support (after disease ontology propagation) have been considered.

| Type of support | Direct | Indirect |
|---|---|---|
| Literature | 302 | 339 |
| Human genetic | 101 | 191 |
| Other non-clinical | 55 | 223 |

**Supplementary Figure S7. Time difference for the same evidence between the primary publication data and the date of deposition into a curated database by an expert curator.** The three data sources that contain evidence with more than one timestamp assigned are shown in the x-axis. Each data point represents an evidence which is mapped to the date difference in the y-axis. Box plots show the median (centre line), the 25th–75th percentiles (box), whiskers extending to the most extreme points within 1.5×IQR, and points beyond whiskers plotted as outliers.

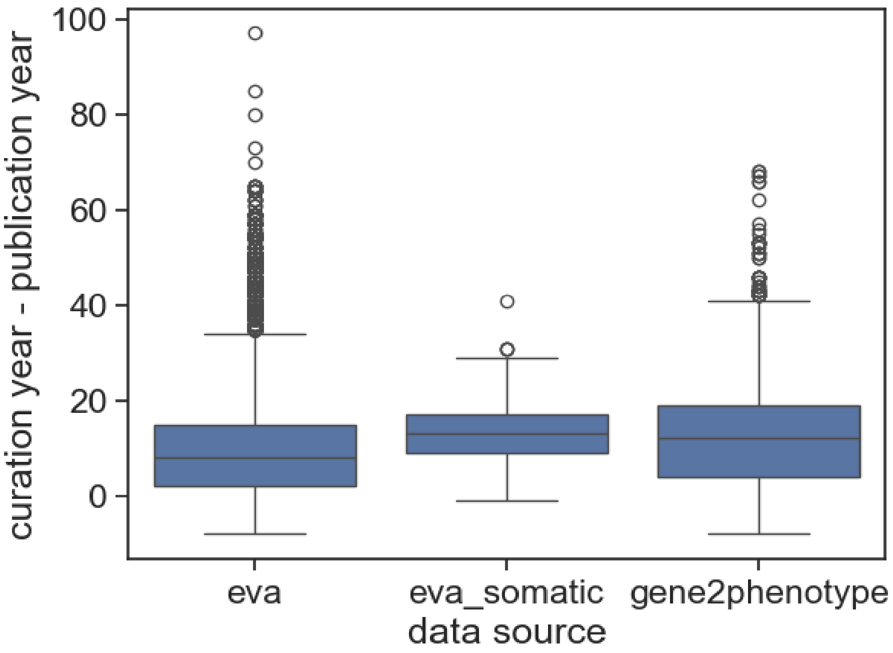

Supplement: Supplementary file 1 — Supplementary Information [file 41467_2025_67180_MOESM1_ESM.pdf]
